# Simulation-based neural dynamics of stress-related negative cognitive bias in depressive symptoms: a dynamical EEG/ERP framework

**DOI:** 10.3389/fnhum.2026.1972914

**Published:** 2026-10-29

**Authors:** Yimin Zhang, Jianping Liu

**Affiliations:** School of Psychology, Jiangxi Normal University, Nanchang, Jiangxi, China

**Keywords:** cognitive bias, depressive symptoms, EEG, event-related potentials, psychological stress

## Abstract

**Introduction:**

This simulation-based computational study develops a non-linear state-space framework for stress-related negative cognitive bias and multivariate EEG/ERP observations.

**Methods:**

A three-state model represents negative bias, cognitive control, and arousal, while symptom load and normalized model stress input reshape a quartic bias potential. N2, P3, late positive potential, frontal theta, frontal alpha asymmetry (FAA), and frontoparietal phase locking provide complementary observation channels. Additional checks distinguish the non-linear benchmark from additive and interaction-only competing generators, examine equilibria of the full coupled system, and perturb the observation model.

**Results:**

We establish bounded stochastic dynamics, a conditional scalar multistability boundary, stable-branch stress sensitivity, and a sufficient local state-observability condition. A seeded benchmark with 180 simulated participants yields model-implied stress-by-symptom slopes of 66.83 ms for response time, 1.12 μV for LPP, 0.775 dB for frontal theta, 0.133 for FAA, and 0.0769 for frontoparietal PLV. Under a non-linear generating process, mean held-out RMSE was 0.060 for the pre-specified quartic candidate vs. 0.436 for the interaction-only candidate. State-reconstruction RMSE increased from 0.152 under the nominal loading matrix to 0.232 under 15% loading perturbations.

**Discussion:**

These results demonstrate implementation consistency and characterize model sensitivity; they are not clinical effect estimates or evidence of empirical parameter identifiability.

## 1 Introduction

Depression is not only characterized by persistent negative mood but also involves systematic distortions in attention, interpretation, memory, and self-referential appraisal. Contemporary accounts increasingly treat these distortions as interacting neural computations rather than isolated symptoms. A recent integrative theory argues that negative cognitive bias can emerge from coupled abnormalities in emotional salience, attentional control, rumination, and inhibition (Jiang, 2024). This view is consistent with the broader EEG literature, in which depressive states are associated with alterations in oscillatory activity, asymmetry, connectivity, and task-evoked potentials (Kopańska et al., 2025). However, most electrophysiological studies still summarize each marker independently. Such an approach can identify associations, but it provides only a limited account of how stress pushes a vulnerable cognitive system from flexible processing toward a persistent negative mode.

Stress is treated here as a candidate control parameter for this transition, while its empirical calibration remains a separate question. Event-related potential research shows that acute and naturalistic stress can alter reward responsiveness, particularly the reward positivity, although effect sizes vary across paradigms and samples (Pegg and Kujawa, 2024). Emotional-face EEG studies further demonstrate that major depressive disorder is accompanied by stage-specific changes in functional network topology during affective processing (Teng et al., 2024). Self-referential processing is also clinically informative. In depressed adolescents receiving cognitive behavioral therapy, neurophysiological responses during self-referential processing have been associated with subsequent improvement (Kim et al., 2025). These findings suggest that stress, self-relevance, and symptom severity should not be modeled as additive nuisance variables. They may jointly alter the stability of a latent processing regime that determines how strongly negative information captures attention and remains represented after stimulus onset.

Several ERP components provide temporally resolved windows into this regime. Reduced reward responsiveness has been proposed as a vulnerability process in depression, with social context and intervention implications (Kujawa, 2024). The late positive potential (LPP), which reflects sustained motivated attention to affective material, is sensitive to emotion-regulation difficulty in adolescents with current or familial risk for depression (Dickey et al., 2024). Resting and task EEG additionally point to abnormal alpha regulation in severe depressive phenotypes, including suicidal depression (Zhao et al., 2025). In subthreshold depressive states, frontal-parietal ERPs during emotional stimulation can already show biased processing (Li et al., 2023). A recent meta-analysis also indicates that unipolar depression is associated with reproducible alterations in P300 amplitude and latency, although heterogeneity remains substantial across tasks and recording choices (Santopetro et al., 2025). The common limitation is that N2, P3/P300, LPP, and oscillatory measures are rarely linked through one dynamical mechanism.

Stress-related dysregulation should be interpreted against the broader electrophysiological background of depression. A recent systematic review and meta-analysis of resting-state EEG identified recurring oscillatory abnormalities while also emphasizing substantial heterogeneity across recording and analytic choices (Heitmann et al., 2026). At the same time, social appraisal tasks reveal ERP abnormalities in major depressive disorder that involve distorted evaluation of socio-emotional information (Li et al., 2026). Rumination may further alter how negative material is processed during goal-directed behavior, as suggested by recent ERP evidence (Owens et al., 2025). These observations motivate a model in which stress increases arousal, weakens top-down control, and changes the effective energy landscape of negative appraisal. Importantly, such a model should distinguish a transient state shift from a genuine change in attractor structure. A transient effect returns quickly after stress removal, whereas an attractor change can preserve a negative processing tendency after the external perturbation diminishes.

Measurement reliability is equally important. ERP biomarkers are useful only if individual differences are sufficiently stable across sessions and hardware. Recent precision-EEG work has therefore emphasized reliability at the individual level rather than group-mean significance alone (Korisky et al., 2026). Complementary resting-state evidence shows that theta power, theta-beta ratio, frontal-parietal phase locking, and source-localized activity vary with depressive state even in otherwise healthy individuals (Li et al., 2024). Time-varying EEG network analyses during facial emotion tasks similarly indicate that depression-related differences evolve across processing stages instead of remaining static (Yang et al., 2024). Together, these findings support a multivariate observation model: an unobserved neural bias state should be inferred from several partially redundant EEG/ERP channels, with explicit attention to local state observability, reconstruction error, and sensitivity to missing observables.

Recent 2024 work also strengthens the case for studying stress and positive/negative valence jointly. Interpersonal trauma can interact with neurophysiological responses to positive interpersonal images when accounting for adolescent depression (Long et al., 2024). Methodological choices such as ocular-correction electrode selection can measurably change reward positivity and LPP estimates (Pegg et al., 2024). Delta-beta coupling has been related to depressive symptoms and treatment outcomes in adolescents, further highlighting cross-frequency regulation rather than a single-band biomarker (Venanzi et al., 2024). In adults with major depressive disorder, resting and task EEG differences have been observed across spectral and evoked measures (Kovacevic et al., 2025). Broad reviews of EEG-based depression assessment now converge on the need for better interpretability, validation, and multimarker integration (Elnaggar et al., 2025). Thus, a high-value next step is not simply another classifier, but a mechanistic state-space model that links symptom vulnerability, acute stress, behavior, ERPs, and oscillatory network measures.

Clinical interpretation must nevertheless remain cautious. P300 abnormalities can discriminate depressive groups in some settings, including comparisons between unipolar depression, bipolar II depression, and controls, but they are not disorder-specific signatures (Zhou et al., 2025). Time-resolved multivariate EEG decoding of affective self-referential sentences has revealed altered temporal organization of semantic evaluation in depression and suicidality (Jeong et al., 2026). Complementary resting-state evidence in adolescents with major depression and suicidal ideation indicates alpha-band fronto-limbic hyperconnectivity, underscoring the importance of network context rather than isolated power measures (Zhang et al., 2026). Longitudinal stress-related work further shows that monetary and social reward responsiveness can moderate the association between chronic stress and depressive symptoms in adolescents (Pegg et al., 2026). Related work using the same affective-sentence paradigm also shows that task-evoked EEG responses can carry group-level information about depressive status, but the reported classification performance does not justify treating a single task as a standalone diagnostic biomarker (Kommineni et al., 2026). These findings caution against equating any single waveform, connectivity feature, or band-power measure with a latent psychiatric construct and motivate the constrained multimarker formulation used here.

The present Original Research study investigates these questions computationally using a non-linear latent-state model and a fully reproducible synthetic EEG/ERP experiment; it does not report human EEG. The intended novelty is the integration of four elements rather than the invention of state-space modeling itself: a stress- and symptom-dependent quartic bias coordinate, explicit coupling to control and arousal, a constrained multivariate EEG/ERP observation layer, and falsifiable comparisons against simpler generators. The formulation is informed by frontal-asymmetry work (Davidson, 1998) and source-level evidence linking the LPP to distributed emotional-processing generators (Liu et al., 2012). We ask whether (i) the stochastic dynamics remain bounded; (ii) the conditional scalar subsystem admits a transparent multistability boundary; (iii) the full coupled equilibrium geometry preserves the same qualitative possibility of multistability; (iv) the observation map is locally state-observable and reasonably conditioned; and (v) implementation, competing-generator discrimination, oracle-state coefficient recovery, and observation-model sensitivity behave as expected under controlled simulation. These objectives deliberately separate mathematical observability, state reconstruction, coefficient recovery with oracle latent states, and empirical parameter identifiability. The resulting study is evidence about the mathematical and numerical behavior of the proposed model, not about clinical effect sizes in a human population. Table 1 summarizes the principal notation.

**Table 1:** Main symbols and definitions.

Symbol	Definition	Symbol	Definition
*t*	Continuous neural-processing time	*d*∈[0, 1]	Depressive symptom load
*s*(*t*)	Normalized stress input	*v*∈{−1, 0, 1}	Stimulus valence code
*b*(*t*)	Latent negative-bias coordinate	*c*(*t*)	Cognitive-control coordinate
*r*(*t*)	Arousal coordinate	*z*(*t*)	Joint latent state (*b, c, r*)^⊺^
*V*(*b*; *d, s, c*)	Conditional stress-bias potential	*a*(*d, s*)	Quadratic landscape coefficient
*h*(*d, s*)	Directional bias field	κ	Quartic confinement coefficient
γ	Control-to-bias coupling	λ_*c*_	Control relaxation rate
λ_*r*_	Arousal relaxation rate	σ_*b*_	Bias-state diffusion scale
*y*_*k*_	EEG/ERP observation vector	*H*_*k*_	Observation loading matrix
*R*	Observation-noise covariance	*W*_*o*_	Local observability Gramian
*A*_*ij*_	Functional connectivity weight	*P*_θ_	Frontal theta power
FAA	Frontal alpha asymmetry	PLV_*ij*_	Phase-locking value
Δ*V*	Potential-barrier height	τ_esc_	Mean attractor escape time

## 2 Materials and methods

### 2.1 Latent stress-bias state

We represent cognitive processing during an emotional trial by the latent state in Equation 1:

$$\begin{array}{l} z\left(t\right)={\left[\begin{array}{ccc} b\left(t\right) & c\left(t\right) & r\left(t\right) \end{array}\right]}^{⊤}\in {\mathbb{R}}^{3}, \end{array}$$ (1)

where *b* is a signed negative-bias coordinate, *c* summarizes control available for disengagement and reappraisal, and *r* is a non-specific arousal coordinate. Positive *b* means that negative information receives disproportionate processing weight. In the present simulations, *d*∈[0, 1] is a unitless severity index used to span the model's trait dimension; it is not a standardized questionnaire score or an estimated clinical latent trait. A future empirical application should specify a preregistered transformation from a named symptom instrument to *d*. Similarly, *s*(*t*) is a normalized model input representing stress load. It should not be equated with a real experimental manipulation until a manipulation check and calibration model link the laboratory stressor to this latent input. The symbols λ_*c*_ and λ_*r*_ below are relaxation rates, not stress variables. Their non-linear interaction is encoded by the curvature coefficient in Equation 2:

$$\begin{array}{l} a\left(d,s\right)=a_{0}+a_{d}d+a_{s}s+a_{ds}ds, \end{array}$$ (2)

and the directional field in Equation 3:

$$\begin{array}{l} h\left(d,s\right)=h_{0}+h_{d}d+h_{s}s+h_{ds}ds. \end{array}$$ (3)

The coefficient *a* changes the curvature of the state landscape, while *h* tilts the landscape toward or away from the negative attractor. This separation is useful: an increase in *h* shifts the preferred state, whereas an increase in *a* can create bistability and hence path dependence.

We define the one-dimensional bias potential conditional on control in Equation 4:

$$\begin{array}{l} V\left(b;d,s,c\right)=\frac{\kappa }{4}b^{4}-\frac{a\left(d,s\right)}{2}b^{2}-[h\left(d,s\right)-\gamma c]b, \end{array}$$ (4)

with *κ* > 0. The deterministic bias flow is the negative potential gradient,

$$\begin{array}{l} ḃ=-\frac{\partial V}{\partial b}=a\left(d,s\right)b-\kappa b^{3}+h\left(d,s\right)-\gamma c. \end{array}$$ (5)

Control and arousal are coupled through

$$\begin{array}{l} ċ=-\lambda_{c}\left(c-c_{0}\right)-\rho r-\eta b+u_{c}\left(t\right), \end{array}$$ (6)

The arousal dynamics are given by Equation 7:

$$\begin{array}{l} ṙ=-\lambda_{r}r+\chi s\left(t\right)+\omega |v\left(t\right)|+u_{r}\left(t\right), \end{array}$$ (7)

where *u*_*c*_ and *u*_*r*_ permit task-specific inputs. The terms −ρ*r* and −η*b* encode two routes by which heightened arousal and sustained negative processing can reduce effective regulatory control. Conversely, −γ*c* in Equation 5 allows stronger control to oppose the directional pull toward negative processing. The resulting proposed generative architecture is summarized in Figure 1.

**Figure 1:**
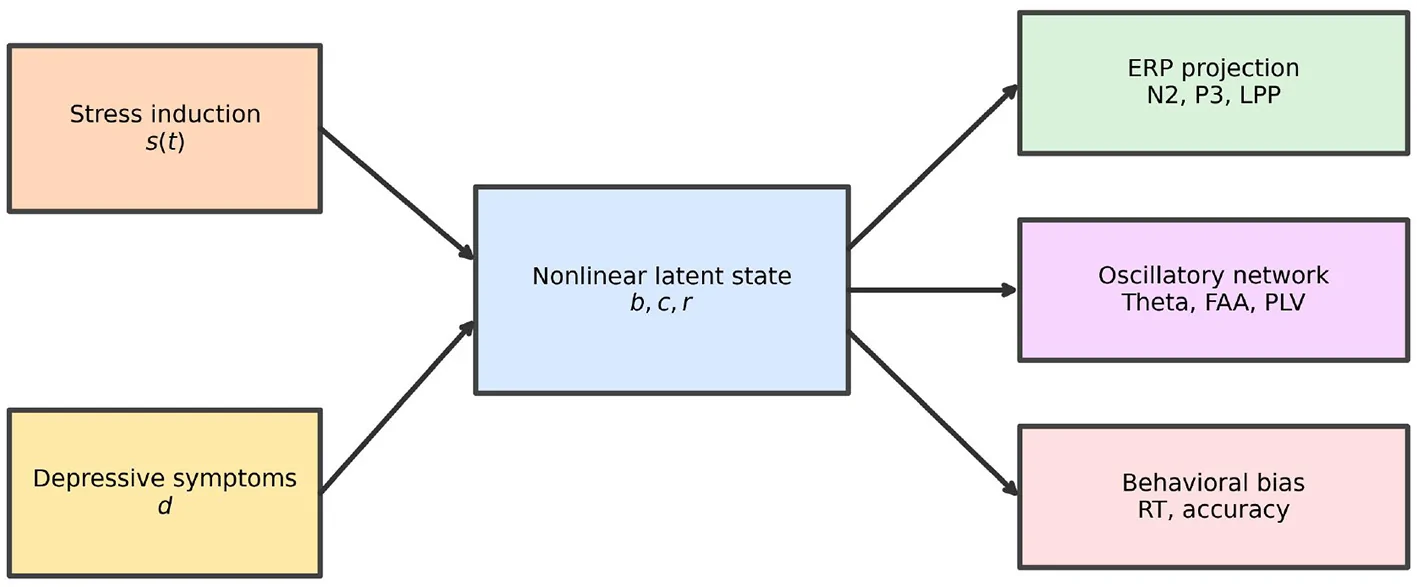
Conceptual architecture of the stress-bias neural dynamics framework.

Figure 1 summarizes the hypothesized mechanistic organization specified by the model; the directed arrows are generative assumptions rather than empirically identified causal effects. Stress and symptom load enter the latent system through distinct but interacting parameters, rather than being collapsed into one severity score. The latent coordinates then project to ERP amplitudes, oscillatory-network measures, and behavior. This separation makes the framework falsifiable: the same inferred bias trajectory must explain several observables at once. A model that fits LPP while failing to reproduce control-sensitive P3 or frontoparietal connectivity is therefore rejected rather than rescued by *post-hoc* component-specific narratives.

### 2.2 Stochastic neural dynamics

Trial-to-trial neural variability is represented by an Itô stochastic differential equation. Let ***W***_*t*_ be a three-dimensional Wiener process. Then,

$$\begin{array}{l} dz=f\left(z;d,s,v\right)dt+GdW_{t}, \end{array}$$ (8)

with the drift in Equation 9:

$$\begin{array}{l} f=\left[\begin{array}{c} ab-\kappa b^{3}+h-\gamma c\\ -\lambda_{c}\left(c-c_{0}\right)-\rho r-\eta b\\ -\lambda_{r}r+\chi s+\omega |v| \end{array}\right], \end{array}$$ (9)

and the diffusion matrix in Equation 10:

$$\begin{array}{l} G=\mathrm{diag}\left(\sigma_{b},\sigma_{c},\sigma_{r}\right). \end{array}$$ (10)

For the scalar bias coordinate with slowly varying c, the Fokker-Planck equation is given by Equation 11:

$$\begin{array}{l} \frac{\partial p}{\partial t}=\frac{\partial }{\partial b}\left(p\frac{\partial V}{\partial b}\right)+\frac{\sigma_{b}^{2}}{2}\frac{\partial^{2}p}{\partial b^{2}}. \end{array}$$ (11)

Its zero-flux stationary density is given by Equation 12:

$$\begin{array}{l} p_{\infty }\left(b|d,s,c\right)=\frac{1}{Z}\exp\left[-\frac{2V\left(b;d,s,c\right)}{\sigma_{b}^{2}}\right], \end{array}$$ (12)

where the normalization constant is given by Equation 13:

$$\begin{array}{l} Z=\int_{-\infty }^{\infty }\exp\left[-\frac{2V\left(u;d,s,c\right)}{\sigma_{b}^{2}}\right]du. \end{array}$$ (13)

Thus, a deeper negative well increases both occupancy probability and persistence without requiring an arbitrarily large linear coefficient.

**Theorem 1** (Bounded moments and invariant measure). *Assume κ*>0*, λ*_*c*_>0*, λ*_*r*_>0*, bounded inputs s*(*t*) *and v*(*t*), *and finite diffusion coefficients. Then, the process in *Equation 8* is non-explosive. Moreover, for constant inputs, it admits at least one invariant probability measure with finite second moments*.

*Proof*. Consider the Lyapunov function in Equation 14:

$$\begin{array}{l} L\left(z\right)=\frac{1}{2}b^{2}+\frac{q_{c}}{2}c^{2}+\frac{q_{r}}{2}r^{2}, \end{array}$$ (14)

with positive *q*_*c*_, *q*_*r*_. The infinitesimal generator satisfies Equation 15:

$$\begin{array}{ll} AL= & ab^{2}-\kappa b^{4}+\left(h-\gamma c\right)b+q_{c}c\left[-\lambda_{c}\left(c-c_{0}\right)-\rho r-\eta b\right]\\ & +q_{r}r\left[-\lambda_{r}r+\chi s+\omega |v|\right]+\frac{1}{2}\mathrm{tr}\left(G^{⊤}\nabla^{2}LG\right). \end{array}$$ (15)

Apply Young's inequality to every cross term. For arbitrary ϵ_1_>0, Equation 16 gives

$$\begin{array}{l} |\left(h-\gamma c\right)b|\leq \epsilon_{1}b^{4}+C_{1}\left(1+c^{2}\right), \end{array}$$ (16)

and analogous bounds hold for *cr*, *bc*, and bounded input terms. Choose ϵ_1_ < κ/2 and then select *q*_*c*_, *q*_*r*_ such that the negative quadratic terms dominate the remaining cross terms. Consequently, constants *K*_0_, *K*_1_, *K*_2_>0 exist with

$$\begin{array}{l} AL\leq K_{0}-K_{1}b^{4}-K_{2}\left(c^{2}+r^{2}\right). \end{array}$$ (17)

This Foster–Lyapunov drift condition prevents finite-time explosion and yields tightness of empirical occupation measures. Standard Krylov–Bogoliubov arguments then provide an invariant probability measure. Integrating Equation 17 under that measure gives finite second moments.

*Remark*. Theorem **??** is more than a technical safeguard. It ensures that the model cannot explain an extreme ERP observation by letting an unobserved bias state diverge without limit. The quartic term provides non-linear confinement of the latent bias state, while control and arousal have dissipative relaxation. Therefore, large simulated or empirical excursions must be explained through finite parameter changes, altered noise, or switching between attractors rather than numerical instability.

### 2.3 EEG/ERP observation model and synthetic experimental design

#### 2.3.1 Stress-contingent cognitive bias paradigm

The proposed human experiment combines a stress manipulation with emotional self-relevance judgments. Figure 2 presents one trial. Participants would first complete a neutral baseline block and a validated acute-stress block, counterbalanced across individuals. Each trial begins with a context cue, followed by fixation and an emotional face or adjective. Participants judge whether the stimulus is self-relevant or rapidly categorize its valence. Negative, neutral, and positive items are balanced for perceptual complexity and normative arousal. The design is compatible with continuous symptom measurement rather than requiring a categorical diagnosis.

**Figure 2:**
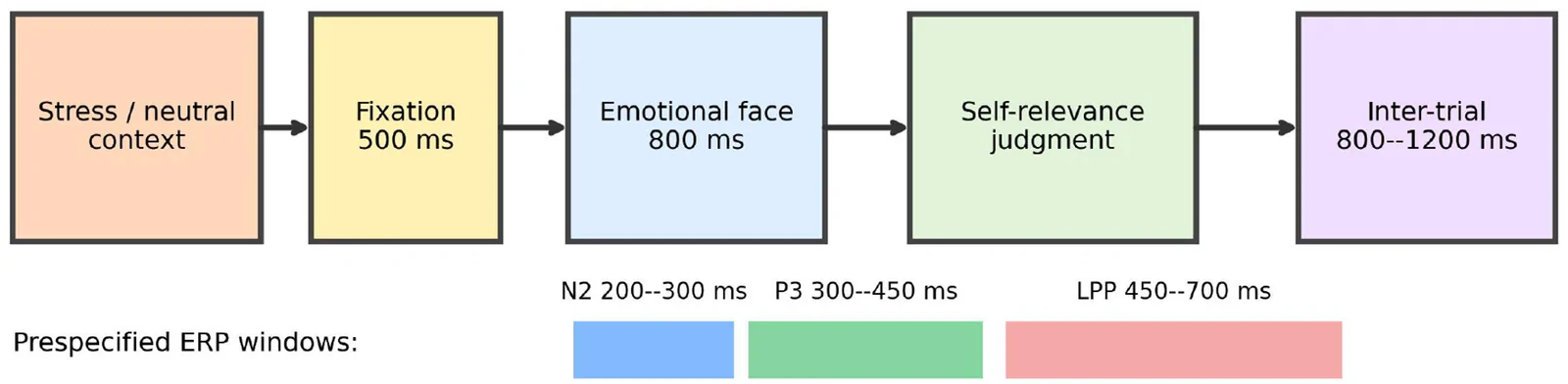
Trial structure and pre-specified ERP windows for the proposed stress-contingent bias paradigm.

Figure 2 separates early conflict-sensitive and later evaluative processes. The N2 window is defined a priori at 200–300 ms, P3 at 300–450 ms, and LPP at 450–700 ms. These windows are not treated as independent tests of unrelated hypotheses. Instead, they constrain the temporal loading matrix of one latent state-space model. The proposed design therefore discourages flexible peak selection after inspection of group differences. It also enables preregistration of electrode clusters, preprocessing, stress manipulation checks, trial exclusions, and the direction of each model-predicted interaction.

#### 2.3.2 EEG pre-processing and feature construction

No channel-level EEG or sampled time series enters the present summary-level generator. The previously stated recommendation of at least 64 scalp channels, 500–1,000 Hz sampling, and synchronized event markers refers only to a proposed future empirical protocol; neither the channel count nor the sampling rate is a numerical input to the current synthetic benchmark. Future recordings would be re-referenced, band-pass filtered, segmented, and corrected for ocular artifacts using a pre-specified independent-component procedure. Methodological evidence shows that ocular-correction electrode choices can materially affect ERP estimates (Pegg et al., 2024), and EEGLAB provides a standard computational basis for independent-component EEG processing (Delorme and Makeig, 2004). Figure 3 is separately generated from 32 idealized 10–20 coordinates solely for topographic visualization; it is not meant to represent the proposed acquisition montage. Thus, there is no 32-vs.-64 channel inconsistency in the computation: 32 coordinates are a plotting device, whereas ≥64 channels are a future acquisition recommendation.

**Figure 3:**
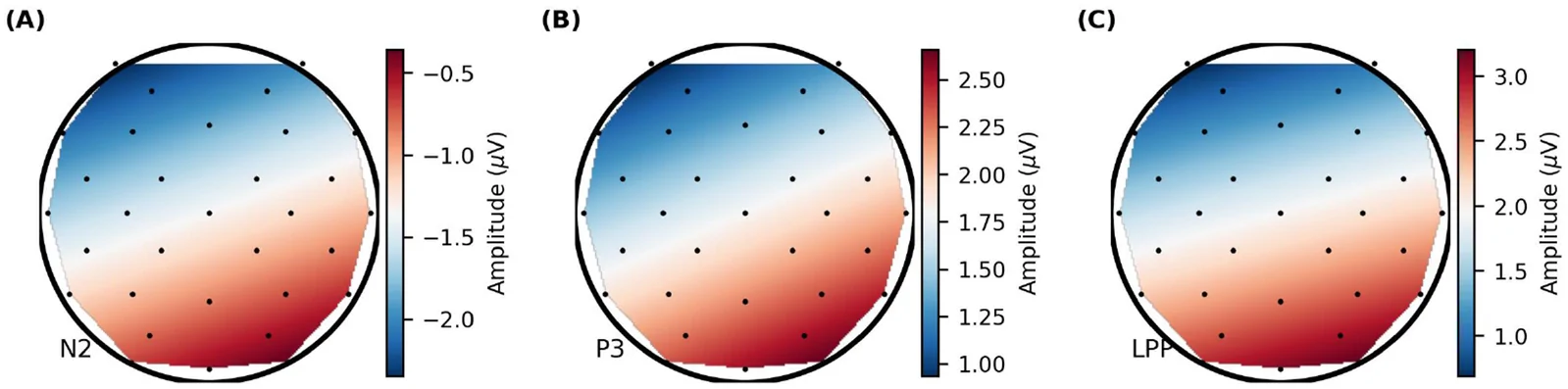
Synthetic scalp fields for **(A)** N2, **(B)** P3, and **(C)** LPP analysis windows.

For trial *k*, let the feature vector be defined by Equation 18:

$$\begin{array}{l} y_{k}={\left[\begin{array}{cccccc} N2_{k} & P3_{k} & LPP_{k} & P_{\theta ,k} & FAA_{k} & PLV_{k} \end{array}\right]}^{⊤}. \end{array}$$ (18)

We model this vector using Equation 19:

$$\begin{array}{l} y_{k}=H_{k}z\left(t_{k}\right)+q\left(v_{k},s_{k},d\right)+\epsilon_{k}, \end{array}$$ (19)

where $\epsilon_{k}\sim N\left(0,R\right)$. A structured loading matrix is

$$\begin{array}{l} H_{k}=\left[\begin{array}{ccc} -\beta_{N2} & 0 & \alpha_{N2}\\ -\beta_{P3} & \alpha_{P3} & -\delta_{P3}\\ \beta_{LPP} & -\delta_{LPP} & \alpha_{LPP}\\ \beta_{\theta } & -\delta_{\theta } & \alpha_{\theta }\\ \beta_{FAA} & 0 & 0\\ \beta_{PLV} & \alpha_{PLV} & 0 \end{array}\right]. \end{array}$$ (20)

The signs encode falsifiable hypotheses: stronger negative bias predicts a more negative N2, lower P3, larger LPP to negative stimuli, increased frontal theta, and altered frontoparietal coupling. Control contributes positively to P3 and coordinated connectivity. Arousal loads most directly on frontal theta and can modestly alter early negativity.

The EEG/ERP observation loading structure in Equation 20 is a theoretically specified model component. Its prescribed signs, zero entries, and loading magnitudes are assumptions of the present synthetic benchmark and require future empirical calibration using human EEG datasets.

A negative-vs.-positive behavioral bias index is defined by Equation 21:

$$\begin{array}{l} B_{RT}=RT_{neg}-RT_{pos}, \end{array}$$ (21)

while accuracy bias is defined by Equation 22:

$$\begin{array}{l} B_{ACC}=Acc_{pos}-Acc_{neg}. \end{array}$$ (22)

For component *m*, the stress modulation index is defined by Equation 23:

$$\begin{array}{l} \Delta_{m}\left(d\right)=𝔼\left[y_{m}|s=1,v=1,d\right]-𝔼\left[y_{m}|s=0,v=1,d\right]. \end{array}$$ (23)

We specifically predict the directional constraints in Equation 24:

$$\begin{array}{l} \frac{\partial \Delta_{LPP}}{\partial d}>0,\;\frac{\partial \Delta_{\theta }}{\partial d}>0,\;\frac{\partial \Delta_{FAA}}{\partial d}>0,\;\frac{\partial \Delta_{P3}}{\partial d}<0. \end{array}$$ (24)

These are model-level directional constraints, not results inferred from the synthetic benchmark.

#### 2.3.3 Oscillatory and network observables

For channel *i*, frontal theta power is estimated from the log spectrum using Equation 25:

$$\begin{array}{l} P_{\theta ,i}=10\log_{10}\left(\frac{\int_{4}^{7}S_{i}\left(f\right)df}{\int_{4}^{7}S_{i}^{base}\left(f\right)df}\right). \end{array}$$ (25)

FAA is defined by Equation 26:

$$\begin{array}{l} FAA=\log P_{\alpha ,F4}-\log P_{\alpha ,F3}. \end{array}$$ (26)

For analytic phases $\phi_{i}^{\left(n\right)}$ and $\phi_{j}^{\left(n\right)}$ over *N* trials, phase-locking value is defined by Equation 27:

$$\begin{array}{l} PLV_{ij}=|\frac{1}{N}\sum_{n=1}^{N}e^{\text{i}\left(\phi_{i}^{\left(n\right)}-\phi_{j}^{\left(n\right)}\right)}|. \end{array}$$ (27)

We then define a frontoparietal summary in Equation 28:

$$\begin{array}{l} PLV_{FP}=\frac{1}{\left|E_{FP}\right|}\sum_{\left(i,j\right)\in E_{FP}}PLV_{ij}. \end{array}$$ (28)

The state-conditioned connectivity matrix is specified in Equation 29:

$$\begin{array}{l} A\left(t\right)=A_{0}+\beta_{b}b\left(t\right)C_{b}+\beta_{c}c\left(t\right)C_{c}+E\left(t\right), \end{array}$$ (29)

where ***C***_*b*_ emphasizes salience/negative-processing edges and ***C***_*c*_ emphasizes control-related edges. This construction links network reconfiguration to the same latent dynamics that generate ERPs.

*Remark*. The observation model deliberately uses heterogeneous markers. An ERP amplitude alone can be shifted by reference choice, latency jitter, or residual artifact. A connectivity measure alone can be influenced by volume conduction and preprocessing. Requiring N2, P3, LPP, theta, asymmetry, PLV, and behavior to agree through a low-dimensional latent trajectory creates cross-measure constraints. In a future empirical test, disagreement among modalities would become evidence against the model rather than an inconvenience to be averaged away.

### 2.4 Synthetic benchmark generation and statistical analysis

The synthetic experiment was designed first as an internal-consistency benchmark and then extended with targeted reviewer-requested structural checks. It contains no recordings from patients, healthy volunteers, or public clinical datasets. We generated *N* = 180 synthetic participants with continuous *d*∈[0, 1]. The labels low, moderate, and elevated are descriptive plotting strata obtained by rank-ordering the simulated *d* values and dividing them into three equal groups of 60; they do not define diagnostic categories or thresholds. Each participant contributed baseline (*s* = 0) and benchmark stress (*s* = 1) conditions and negative, neutral, and positive valence summaries, yielding 1,080 rows. The random seed is 20260819. All outputs are regenerated by code/run_all.py. The primary summary-data benchmark uses a reduced quasistatic generator rather than time integration of Equation 8; separate full-coupled equilibrium calculations are reported below to test whether the scalar multistability geometry survives restoration of *b*–*c*–*r* feedback. The main benchmark settings are summarized in Table 2.

**Table 2:** Synthetic benchmark design and generative assumptions.

Item	Setting	Item	Setting
Synthetic participants	180; 60 per symptom stratum	Conditions	Baseline and stress
Valence levels	Negative, neutral, positive	Summary rows	1,080
Symptom score	Continuous *d*∈[0, 1]	Stress code	*s*∈{0, 1}
Bias confinement	κ = 1.00	Symptom curvature	*a*_*d*_ = 0.48
Stress curvature	*a*_*s*_ = 0.58	Interaction curvature	*a*_*ds*_ = 0.42
Directional symptom field	*h*_*d*_ = 0.28	Directional stress field	*h*_*s*_ = 0.32
Directional interaction	*h*_*ds*_ = 0.38	Control coupling	γ = 0.30
Primary observables	N2, P3, LPP, theta, FAA, PLV	Behavioral observables	RT and accuracy
Strata definition	Rank-based thirds of simulated *d*; descriptive only	Acquisition inputs	No channel count or sampling rate used
Scalp-map montage	32 idealized coordinates for Figure 3 only	Full-system control reference	*c*_0_ = 0.78 in coupled-equilibrium check

Table 2 fixes the main synthetic assumptions before any result is examined. The parameterization intentionally creates a stress-by-symptom interaction but leaves substantial subject- and observation-level noise. Its purpose is not to mimic a specific published cohort. Rather, it creates a transparent reference case in which the proposed non-linear mechanism is known to be true. This permits tests of mathematical implementation, competing-generator discrimination, oracle-state coefficient recovery, observation-model perturbation, and visualization code; none of these by itself establishes validity in human EEG. Researchers should replace these values with preregistered priors or estimates from real EEG data in future work.

Synthetic summary observables were generated from the latent states with independent subject intercepts and Gaussian residual terms. At the condition-valence summary level, behavioral response time is represented schematically by Equation 30:

$$\begin{array}{l} RT=\beta_{0}+\beta_{s}s+\beta_{d}d+\beta_{v}v_{+}+\beta_{sdv}sdv_{+}+\beta_{b}b+u_{i}+\epsilon_{RT}, \end{array}$$ (30)

where *v*_+_ = max(*v*, 0). A corresponding ERP generator is given by Equation 31:

$$\begin{array}{l} y_{m}=\alpha_{m0}+\alpha_{ms}s+\alpha_{md}d+\alpha_{mv}v_{+}+\alpha_{msdv}sdv_{+}+h_{m}^{⊤}z+u_{im}+\epsilon_{im}. \end{array}$$ (31)

For the interaction check, negative trials are first differenced within participant. For participant *i*,

$$\begin{array}{l} \Delta y_{i}=y_{i,s=1}-y_{i,s=0}=\alpha_{0}+\alpha_{d}d_{i}+\epsilon_{i}. \end{array}$$ (32)

In the balanced two-condition design, α_*d*_ is the stress-by-symptom interaction slope while respecting the paired structure. Because the data are synthetic and the interaction is part of the generator, the repeated-sampling interval quantifies numerical sampling uncertainty within the benchmark rather than population-level clinical uncertainty.

### 2.5 Oracle-state coefficient recovery and missing-observable analyses

We next perform an oracle-state coefficient-recovery check. Here, the true simulated bias coordinate *b* and a noisy version of its derivative are supplied directly to the regression. This is an implementation diagnostic, not an end-to-end EEG parameter-identification experiment and not evidence of structural or practical parameter identifiability from scalp observations. For this isolated check, control is held fixed, so the constant term −γ*c* is absorbed into the intercept *h*_0_. The drift is expressed in Equation 33:

$$\begin{array}{l} ḃ=\left(a_{0}+a_{d}d+a_{s}s+a_{ds}ds\right)b-\kappa b^{3}+h_{0}+h_{d}d+h_{s}s+h_{ds}ds+\epsilon , \end{array}$$ (33)

the drift is linear in the unknown parameter vector conditional on sampled (*b, d, s*). Define the design row in Equation 34:

$$\begin{array}{l} x=\left[b,db,sb,dsb,-b^{3},1,d,s,ds\right], \end{array}$$ (34)

and the parameter vector in Equation 35:

$$\begin{array}{l} \theta ={\left[a_{0},a_{d},a_{s},a_{ds},\kappa ,h_{0},h_{d},h_{s},h_{ds}\right]}^{⊤}. \end{array}$$ (35)

Assuming ***X*** has full column rank, the least-squares estimate is given by Equation 36:

$$\begin{array}{l} \hat{\theta }={\left(X^{⊤}X\right)}^{-1}X^{⊤}\overset{•}{b}. \end{array}$$ (36)

For the missing-observable analysis, latent bias was predicted from subsets of the six pre-specified EEG/ERP observables (N2, P3, LPP, frontal theta, FAA, and frontoparietal PLV). Negative-trial rows were split at the participant level, with 75% of synthetic participants used for training and 25% for testing so that repeated rows from one participant could not cross the split. For each retained-observable count from six to two, 80 randomized repetitions were performed. Predictors were standardized using training-set statistics, an ordinary least-squares observation model was fitted on the training participants, and test-set latent-bias RMSE was recorded. This procedure tests state-reconstruction degradation under missing observables without information leakage across participants; it does not test dynamic-parameter identifiability.

### 2.6 Competing-generator, coupled-equilibrium, and observation-sensitivity checks

Three additions were made to separate implementation verification from broader model evaluation. First, 100 train/test repetitions were generated under each of three data-generating structures: an additive linear model, a linear stress-by-symptom interaction, and the pre-specified quartic-equilibrium benchmark. Each synthetic dataset contained 600 (*d, s*) points with *s*∈[0, 1.4] and Gaussian noise SD 0.06. Candidate additive and interaction regressions were fitted on 400 points and evaluated on 200 held-out points. The quartic candidate used the pre-specified equilibrium curve from Equations 38–42, with only an intercept and scale calibration estimated in the training data. This check asks whether the benchmark can be distinguished from simpler alternatives when its structural parameters are pre-specified; it is not a claim of general model superiority.

Second, equilibrium geometry was recomputed for the full deterministic coupled system at constant stress and *v* = 0. Setting the control and arousal derivatives to zero gives Equation 37:

$$\begin{array}{l} r^{*}=\frac{\chi s}{\lambda_{r}},\;c^{*}=c_{0}-\frac{\rho r^{*}+\eta b^{*}}{\lambda_{c}}. \end{array}$$ (37)

Substitution into the bias equation yields a feedback-modified cubic. Every real root was evaluated using the eigenvalues of the full 3 × 3 Jacobian; an equilibrium was labeled locally stable only when all eigenvalues had negative real parts. This directly checks the scope of the scalar fixed-control result rather than assuming that its stability classification transfers unchanged to the coupled system.

Third, observation-model sensitivity was evaluated with a standardized 6 × 3 loading matrix matching the sign pattern in Equation 20. Latent states were reconstructed by regularized least squares from noisy observables over 100 Monte Carlo repetitions. We examined the nominal model, FAA deletion, 15% loading perturbations, added cross-loadings, and correlated observation noise (ρ = 0.30). The condition number of the nominal loading matrix was also recorded. This reconstruction experiment assumes the state-space form and therefore evaluates sensitivity to observation misspecification; it does not estimate dynamic parameters from empirical EEG.

## 3 Results

### 3.1 Attractor geometry and multistability

At an instantaneous equilibrium with fixed *c*, Equation 5 gives

$$\begin{array}{l} \kappa b^{*3}-a\left(d,s\right)b^{*}-\tilde{h}\left(d,s,c\right)=0, \end{array}$$ (38)

where

$$\begin{array}{l} \tilde{h}\left(d,s,c\right)=h\left(d,s\right)-\gamma c. \end{array}$$ (39)

After division by κ, the cubic has the canonical form *b*^3^+*pb*+*q* = 0 with

$$\begin{array}{l} p=-\frac{a}{\kappa },\;q=-\frac{\tilde{h}}{\kappa }. \end{array}$$ (40)

The standard discriminant of the depressed cubic is

$$\begin{array}{l} \Delta_{c}=-4p^{3}-27q^{2}=\frac{4a^{3}-27\kappa {\tilde{h}}^{2}}{\kappa^{3}}. \end{array}$$ (41)

Hence, three distinct real stationary points exist when

$$\begin{array}{l} \Delta_{c}>0\;\Leftrightarrow \;4a^{3}>27\kappa {\tilde{h}}^{2}. \end{array}$$ (42)

Two are local minima, and one is a local maximum whenever *a*>0. Figure 4 illustrates how stress and symptom load alter the potential in a representative fixed-control slice with *c* = 0.55.

**Figure 4:**
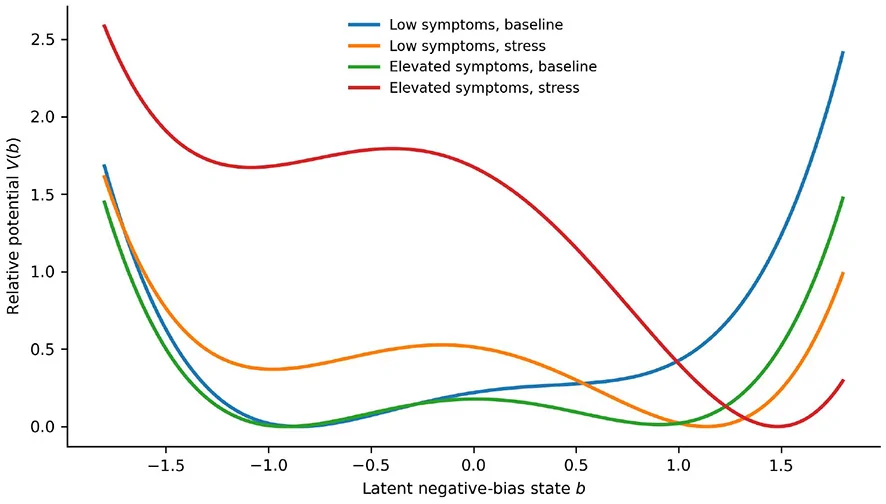
Potential landscapes across symptom load and stress conditions at fixed control.

Figure 4 visualizes the central theoretical claim for the conditional bias potential at *c* = 0.55. Under low symptom load, the potential is comparatively shallow and the negative state is less dominant. Increasing stress tilts and reshapes the landscape, whereas high symptom load magnifies this effect. The important quantity is not simply the location of the minimum. Stress can also reduce the barrier separating flexible and negative-biased regimes, making noise-driven transitions more likely. This provides a mechanistic interpretation of why similar external stressors may yield brief effects in some individuals but persistent negative processing in others.

**Theorem 2** (Multistability criterion). *For fixed d, s, c with a*(*d, s*)>0*, the bias dynamics have two locally asymptotically stable equilibria and one unstable equilibrium if and only if *Equation 42* holds. At equality, a saddle-node boundary is reached*.

*Proof*. Stationary points are the real roots of Equation 38. The discriminant in Equation 41 is positive precisely when $4a^{3}>27\kappa {\tilde{h}}^{2}$, which yields three distinct real roots *b*_1_<*b*_2_<*b*_3_. Linearizing Equation 5 gives the scalar Jacobian in Equation 43:

$$\begin{array}{l} J_{b}\left(b^{*}\right)=a-3\kappa b^{*2}=-V^{\prime \prime }\left(b^{*}\right). \end{array}$$ (43)

Because a quartic potential with positive leading coefficient alternates minimum-maximum-minimum across three simple critical points, $V^{\prime \prime }\left(b_{1}\right)>0$, $V^{\prime \prime }\left(b_{2}\right)<0$, and $V^{\prime \prime }\left(b_{3}\right)>0$. Therefore *J*_*b*_(*b*_1_) < 0 and *J*_*b*_(*b*_3_) < 0, so the outer equilibria are locally asymptotically stable, whereas *J*_*b*_(*b*_2_)>0 makes the middle point unstable. When equality holds, two roots coalesce and *V*″ = 0, producing the saddle-node boundary.

### 3.2 Stress sensitivity on a conditional stable branch

For the conditional scalar bias subsystem, let control follow a differentiable prescribed profile *c* = *c*(*s*); the fixed-control case is recovered with *dc*/*ds* = 0. The model then yields an explicit slope for a stable bias branch. Differentiating $F\left(b^{*},d,s,c\right)=\kappa b^{*3}-ab^{*}-\tilde{h}=0$ with respect to stress gives

$$\begin{array}{l} \frac{db^{*}}{ds}=\frac{a_{s}^{eff}b^{*}+h_{s}^{eff}}{3\kappa b^{*2}-a}, \end{array}$$ (44)

where the effective curvature derivative is given by Equation 45:

$$\begin{array}{l} a_{s}^{eff}=a_{s}+a_{ds}d, \end{array}$$ (45)

and the effective field derivative is given by Equation 46:

$$\begin{array}{l} h_{s}^{eff}=h_{s}+h_{ds}d-\gamma \frac{dc}{ds}. \end{array}$$ (46)

For a stable branch, the denominator is positive because *V*″(b^*^)>0.

** Proposition 1** (Positive stress sensitivity on a stable branch). Let b^*^>0 *lie on a stable branch. If *$a_{s}^{eff}=a_{s}+a_{ds}d\geq 0$* and *$h_{s}^{eff}=h_{s}+h_{ds}d-\gamma \left(dc/ds\right)>0$, then db^*^/*ds*>0*. Moreover, symptom load amplifies this sensitivity whenever the symptom-induced increase in the numerator of *Equation 44* exceeds the proportional increase in the stable-curvature denominator*.

*Proof*. On a stable branch, define the numerator and denominator in Equation 47:

$$\begin{array}{l} N=a_{s}^{eff}b^{*}+h_{s}^{eff},\;D=3\kappa b^{*2}-a>0. \end{array}$$ (47)

The assumptions give *N*>0, and Equation 44 therefore yields db^*^/*ds* = *N*/*D*>0. For symptom moderation, the numerator derivative is given by Equation 48:

$$\begin{array}{l} N_{d}=a_{ds}b^{*}+a_{s}^{eff}\frac{\partial b^{*}}{\partial d}+h_{ds}-\gamma \frac{\partial^{2}c}{\partial d\partial s}, \end{array}$$ (48)

and the curvature derivative is given by Equation 49:

$$\begin{array}{l} D_{d}=6\kappa b^{*}\frac{\partial b^{*}}{\partial d}-\left(a_{d}+a_{ds}s\right). \end{array}$$ (49)

Therefore, the mixed sensitivity is given by Equation 50:

$$\begin{array}{l} \frac{\partial }{\partial d}\left(\frac{db^{*}}{ds}\right)=\frac{N_{d}D-ND_{d}}{D^{2}}. \end{array}$$ (50)

Hence, symptom load amplifies stress sensitivity whenever *N*_*d*_*D*>*ND*_*d*_. This condition is sufficient and directly separates increased stress drive in the numerator from increased local curvature in the denominator. For an equilibrium branch of the full coupled (*b, c, r*) system, the corresponding sensitivity must instead be obtained by simultaneously differentiating all three equilibrium equations.

In the weak-noise, quasi-static limit of the conditional scalar subsystem, the mean escape time from a local minimum *b*_*m*_ across a barrier at *b*_*u*_ is approximated by the overdamped Kramers expression in Equation 51:

$$\begin{array}{l} \tau_{esc}\approx \frac{2\pi }{\sqrt{V^{\prime \prime }\left(b_{m}\right)|V^{\prime \prime }\left(b_{u}\right)|}}\exp\left(\frac{2\Delta V}{\sigma_{b}^{2}}\right), \end{array}$$ (51)

where the potential-barrier height is defined by Equation 52:

$$\begin{array}{l} \Delta V=V\left(b_{u}\right)-V\left(b_{m}\right). \end{array}$$ (52)

Thus, stress can increase negative-state persistence either by shifting the minimum or by reducing the barrier to entry while increasing the barrier to exit. The corresponding equilibrium geometry is visualized in Figure 5.

**Figure 5:**
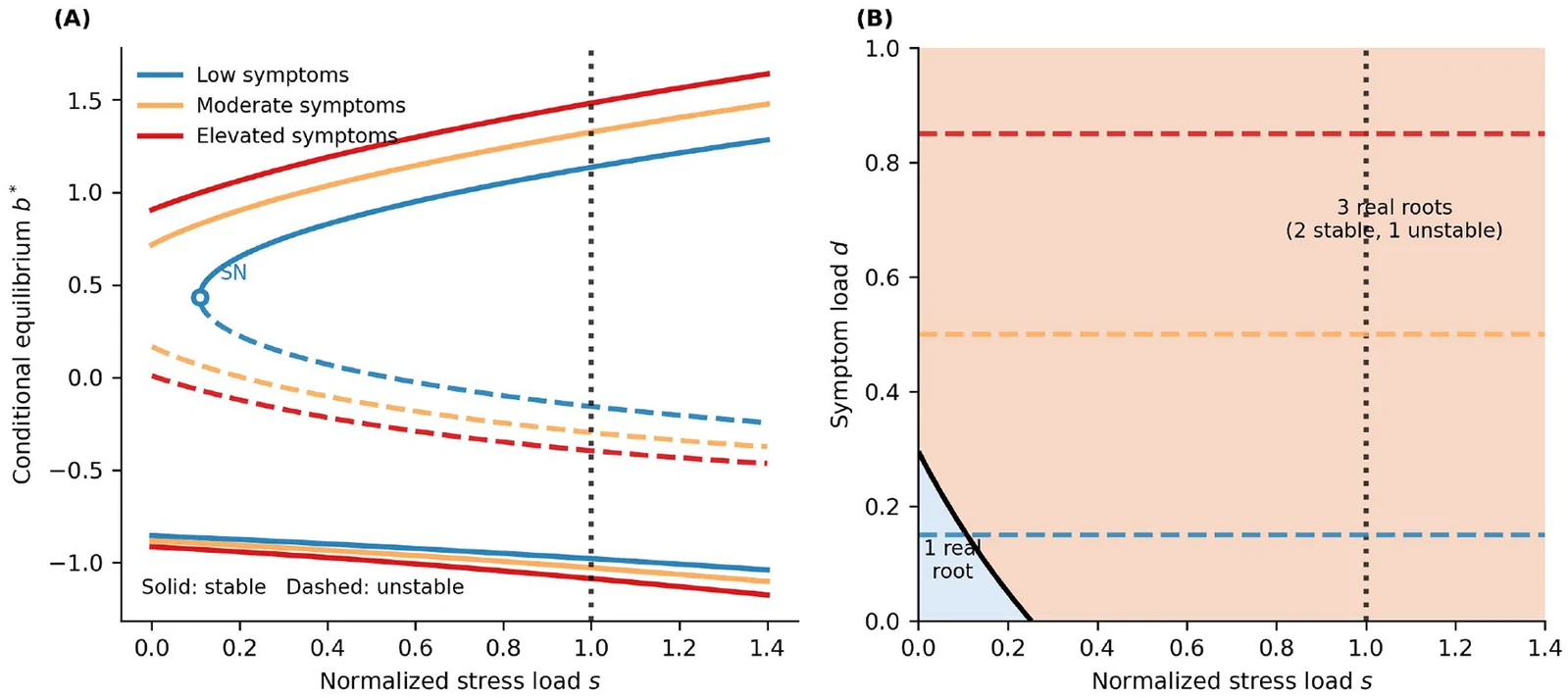
Fixed-control equilibrium branches **(A)** and multistability boundary **(B)** across stress and depressive symptom load.

Figure 5 is drawn directly from Equation 38 and the exact discriminant condition in Equation 42. It is a conditional scalar-bias slice with control fixed at *c* = 0.55, not the equilibrium diagram of the full coupled (*b, c, r*) system. The left panel shows every real root of the cubic for three symptom loads; solid segments satisfy *V*″(b^*^)>0 and are locally stable in the fixed-control bias subsystem, whereas dashed segments are unstable. The right panel classifies the stress–symptom plane into one-real-root and three-real-root regions, with the black curve marking $4a^{3}=27\kappa {\tilde{h}}^{2}$. For the low-symptom slice (*d* = 0.15), the marked saddle-node occurs at *s*≈0.109; the moderate and elevated slices are already in the three-root region at *s* = 0 for this parameterization. Thus, the figure shows where a stable-unstable pair appears or disappears, rather than treating a switch in the globally deepest well as a saddle-node event. In this parameter slice, the lower stable branch can persist after a second stable branch emerges, so movement between wells may remain noise- or history-dependent. This distinction is essential for interpreting future hysteresis or recovery experiments. A fixed-control sensitivity check varied *c* from 0.45 to 0.65. For *d* = 0.15, the stress value at which the three-root region first appeared shifted from 0.063 (*c* = 0.45) to 0.109 (*c* = 0.55) and 0.154 (*c* = 0.65). For *d* = 0.50 and 0.85, three roots were already present at *s* = 0 throughout this control range. Thus, the exact boundary moves with control, but the qualitative geometry shown in Figure 5 is not specific to *c* = 0.55.

### 3.3 Local state observability

Let $\bar{z}\left(t\right)$ be a nominal trajectory. The local linearization is given by Equation 53:

$$\begin{array}{l} \delta \overset{•}{z}=F\left(t\right)\delta z, \end{array}$$ (53)

with the Jacobian in Equation 54:

$$\begin{array}{l} F\left(t\right)=\left[\begin{array}{ccc} a-3\kappa b^{2} & -\gamma & 0\\ -\eta & -\lambda_{c} & -\rho \\ 0 & 0 & -\lambda_{r} \end{array}\right]. \end{array}$$ (54)

Let the state-transition matrix **Φ**(*t, t*_0_) solve Equation 55:

$$\begin{array}{l} \frac{d}{dt}\Phi \left(t,t_{0}\right)=F\left(t\right)\Phi \left(t,t_{0}\right),\;\Phi \left(t_{0},t_{0}\right)=I. \end{array}$$ (55)

For continuous observations with covariance ***R***, define the observability Gramian in Equation 56:

$$\begin{array}{l} W_{o}\left(t_{0},T\right)=\int_{t_{0}}^{T}\Phi^{⊤}\left(t,t_{0}\right)H^{⊤}\left(t\right)R^{-1}H\left(t\right)\Phi \left(t,t_{0}\right)dt. \end{array}$$ (56)

** Theorem 3** (Sufficient condition for local state observability). *Assume **R***≻0* and continuous **F***(*t*) *and **H***(*t*) *on* [*t*_0_, *T*]. *If **W***_*o*_(*t*_0_, *T*) *is positive definite and the non-linear remainder of **f** is locally Lipschitz with sufficiently small radius around *$\bar{z}$*, then the initial latent perturbation δz*(*t*_0_) *is locally distinguishable from noise-free multimodal observations on* [*t*_0_, *T*].

*Proof*. In the linearized system, two distinct initial perturbations δ*z*_1_ and δ*z*_2_ produce an output difference with the weighted energy in Equation 57:

$$\begin{array}{ll} E_{y} & =\int_{t_{0}}^{T}{\left\|R^{-1/2}H\left(t\right)\Phi \left(t,t_{0}\right)\left(\delta z_{1}-\delta z_{2}\right)\right\|}^{2}dt\\ & ={\left(\delta z_{1}-\delta z_{2}\right)}^{⊤}W_{o}\left(\delta z_{1}-\delta z_{2}\right). \end{array}$$ (57)

Positive definiteness implies $E_{y}>0$ for every non-zero initial difference; hence, the linearized input–output map is injective. For the non-linear system, write the trajectory difference as the linear solution plus a variation-of-constants remainder. Local Lipschitz continuity bounds that remainder by *Cϵ*‖δ*z*_1_−δ*z*_2_‖ inside a ball of radius ϵ. Because ***W***_*o*_≻0, the linearized output map has a strictly positive minimum gain $\sqrt{\lambda_{\min}\left(W_{o}\right)}$. Choosing the neighborhood so that the non-linear remainder has operator norm smaller than half of this gain preserves injectivity of the local input–output map. Therefore, the initial state is locally observable/distinguishable in that neighborhood. This result concerns the state for a specified local model; it does not establish structural parameter identifiability or practical parameter recovery from noisy EEG.

The corresponding regularized estimator is given by Equation 58:

$$\begin{array}{l} \hat{\Theta }=\underset{\Theta }{arg min}\left\{\sum_{k=1}^{K}{\left\|R^{-1/2}\left(y_{k}-{\hat{y}}_{k}\left(\Theta \right)\right)\right\|}^{2}+\lambda_{\Theta }{\left\|\Theta -\Theta_{0}\right\|}^{2}\right\}, \end{array}$$ (58)

where Θ contains dynamic and observation parameters. Equations 59–61 specify an extended Kalman form for a one-step latent update:

$$\begin{array}{l} {\hat{z}}_{k|k-1}={\hat{z}}_{k-1|k-1}+\Delta tf\left({\hat{z}}_{k-1|k-1}\right), \end{array}$$ (59)

$$\begin{array}{l} K_{k}=P_{k|k-1}H_{k}^{⊤}{\left(H_{k}P_{k|k-1}H_{k}^{⊤}+R\right)}^{-1}, \end{array}$$ (60)

$$\begin{array}{l} {\hat{z}}_{k|k}={\hat{z}}_{k|k-1}+K_{k}\left(y_{k}-H_{k}{\hat{z}}_{k|k-1}-q_{k}\right). \end{array}$$ (61)

These equations make clear why multiple EEG/ERP features are useful: they can improve the conditioning of ***W***_*o*_, especially when one component has low reliability.

*Remark*. The multistability and observability results address different failure modes. The first asks when stress changes the qualitative structure of the latent dynamics. The second asks whether that latent change can be recovered from scalp measurements. A theoretically dramatic bifurcation is scientifically unhelpful if N2, P3, LPP, theta, asymmetry, and connectivity load on the states in a nearly collinear way. Conversely, excellent measurement reliability cannot rescue a model whose latent dynamics have no plausible stability structure.

### 3.4 Synthetic ERP and topographic results

Figure 6 shows synthetic grand-average waveforms at Cz for negative trials in the stress condition. The generator produces a progressively more negative N2, a reduced P3, and a larger sustained LPP as symptom load increases. The waveform is intentionally smooth because it represents a grand average rather than raw EEG. No claim is made that these exact amplitudes or latencies occur in real participants. The numerical result demonstrates the model's cross-component constraint: all three components arise from the same latent bias-control-arousal coordinates and therefore should covary in specific directions across stress and symptom levels.

**Figure 6:**
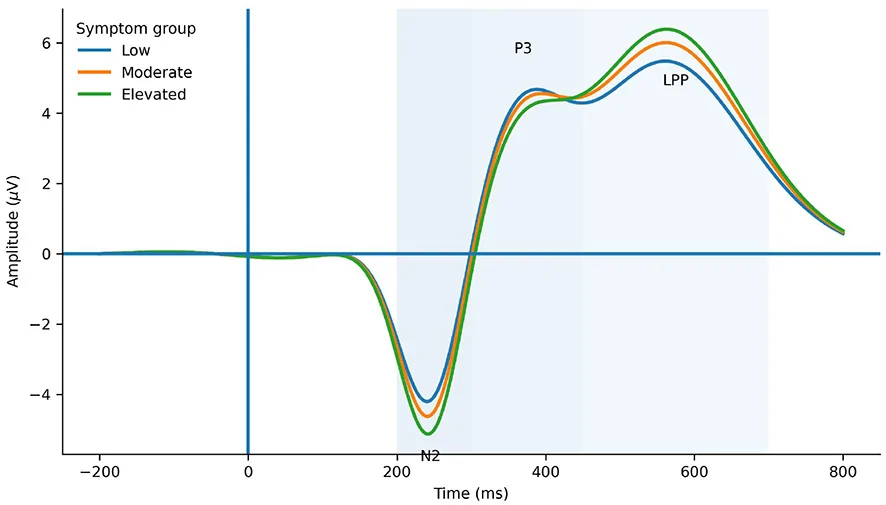
Synthetic stress-condition ERP waveforms for negative stimuli across depressive symptom strata.

Figure 3 displays synthetic scalp fields for the three pre-specified time windows. The N2 field is frontocentrally negative, whereas P3 and LPP show increasingly posterior positive weighting. These maps are generated from idealized 32-channel coordinates solely for visualization and are not inverse solutions; they do not specify the channel count of a future acquisition protocol. Their role is to prevent the observation model from being amplitude-only: future empirical work should test whether the predicted component differences also have plausible topographies. A strong scalar effect with an anatomically inconsistent scalp distribution would indicate artifact, reference dependence, or model misspecification and should weaken the interpretation of a latent bias change.

### 3.5 Stress-by-symptom effects

Table 3 reports model-implied stress-minus-baseline changes for negative trials. The largest response-time shift occurred in the elevated descriptive stratum, 170.83 ms, compared with 132.86 ms in the low stratum. LPP changed by 2.55 μV vs. 1.95 μV, and FAA changed by 0.188 vs. 0.123. Frontal theta and frontoparietal PLV showed the same ordered benchmark pattern. The 95% intervals are repeated-sampling intervals under the specified generator and quantify Monte Carlo/sample variation within this simulation; they are not confidence intervals for a human population or clinical effect sizes.

**Table 3:** Stress-minus-baseline effects for synthetic negative trials.

Symptom stratum	Metric	Mean change	95% lower	95% upper
Low	RT (ms)	132.86	124.93	140.79
Low	N2 (μV)	–1.72	–1.88	–1.57
Low	P3 (μV)	–1.45	–1.60	–1.29
Low	LPP (μV)	1.95	1.75	2.16
Low	Theta (dB)	1.08	1.03	1.14
Low	FAA	0.123	0.105	0.141
Low	Frontoparietal PLV	0.073	0.067	0.079
Moderate	RT (ms)	154.82	148.38	161.27
Moderate	N2 (μV)	–2.04	–2.20	–1.88
Moderate	P3 (μV)	–1.51	–1.67	–1.34
Moderate	LPP (μV)	2.37	2.20	2.54
Moderate	Theta (dB)	1.28	1.22	1.34
Moderate	FAA	0.179	0.162	0.195
Moderate	Frontoparietal PLV	0.087	0.081	0.093
Elevated	RT (ms)	170.83	162.54	179.12
Elevated	N2 (μV)	–2.24	–2.38	–2.10
Elevated	P3 (μV)	–1.65	–1.82	–1.48
Elevated	LPP (μV)	2.55	2.36	2.75
Elevated	Theta (dB)	1.48	1.42	1.54
Elevated	FAA	0.188	0.170	0.205
Elevated	Frontoparietal PLV	0.112	0.105	0.119

Figure 7 visualizes the same generator continuously rather than by descriptive symptom strata and now includes FAA explicitly. Using Equation 32, the model-implied stress-by-symptom slopes were 66.83 ms for RT, 1.12 μV for LPP, 0.775 dB for theta, 0.1326 for FAA, and 0.0769 for PLV. Their 95% repeated-sampling intervals were 46.80–86.85 ms, 0.623–1.619 μV, 0.628–0.922 dB, 0.0874–0.1778, and 0.0605–0.0933, respectively. These values describe the seeded generator and are not neurophysiological effect estimates.

**Figure 7:**
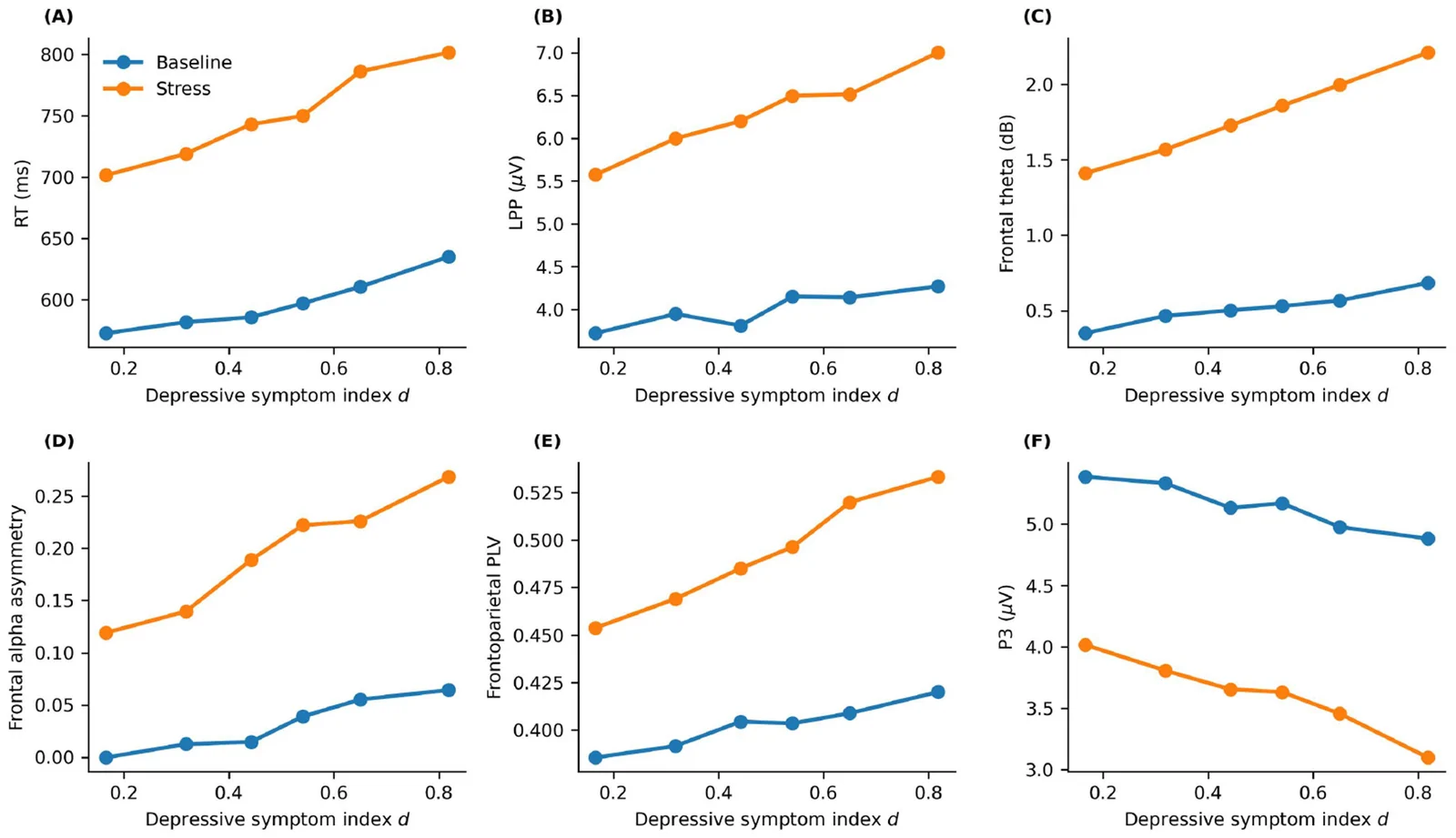
Synthetic model-implied stress-by-symptom patterns for **(A)** RT, **(B)** LPP, **(C)** frontal theta, **(D)** FAA, **(E)** frontoparietal PLV, and **(F)** P3.

### 3.6 Oracle-state coefficient recovery and missing-observable robustness

Table 4 reports 100 Monte Carlo repetitions per derivative-noise level. RMSE increased from 0.0058 at noise SD 0.03 to 0.0267 at SD 0.15, while the ordinary within-replicate regression *R*^2^, averaged across repetitions, remained above 0.99. This result verifies the coefficient-regression implementation when the true latent state and a noisy derivative are supplied; it does not establish structural identifiability, practical parameter identifiability, or end-to-end recovery from EEG observations. Real EEG introduces latent-state estimation error, non-stationarity, correlated artifacts, and imperfect stress manipulation. The table therefore establishes a best-case implementation benchmark. Future empirical work should use hierarchical state-space estimation and report posterior or profile uncertainty rather than relying on direct derivative regression.

**Table 4:** Oracle-state drift-coefficient recovery under increasing derivative noise.

Noise SD	RMSE	MAE	Mean bias	Mean fit *R*^2^
0.03	0.0058	0.0043	0.00005	0.9997
0.06	0.0107	0.0078	0.00009	0.9989
0.10	0.0187	0.0136	0.00002	0.9968
0.15	0.0267	0.0195	0.00023	0.9929

Figure 8 combines recovery and missing-observable stress tests. The left panel uses participant-level train/test splits, so observations from the same synthetic participant never appear in both sets. With all six observables retained, latent-bias RMSE averaged 0.339; with only two observables, it rose to 0.475. The right panel shows that parameter-recovery error increases monotonically with derivative noise. The qualitative message is that redundancy is valuable: no single ERP component is required to carry the complete construct. This benchmark motivates multimodal preregistration, component-deletion sensitivity analyses, and explicit reporting of which observables drive state observability and reconstruction quality.

**Figure 8:**
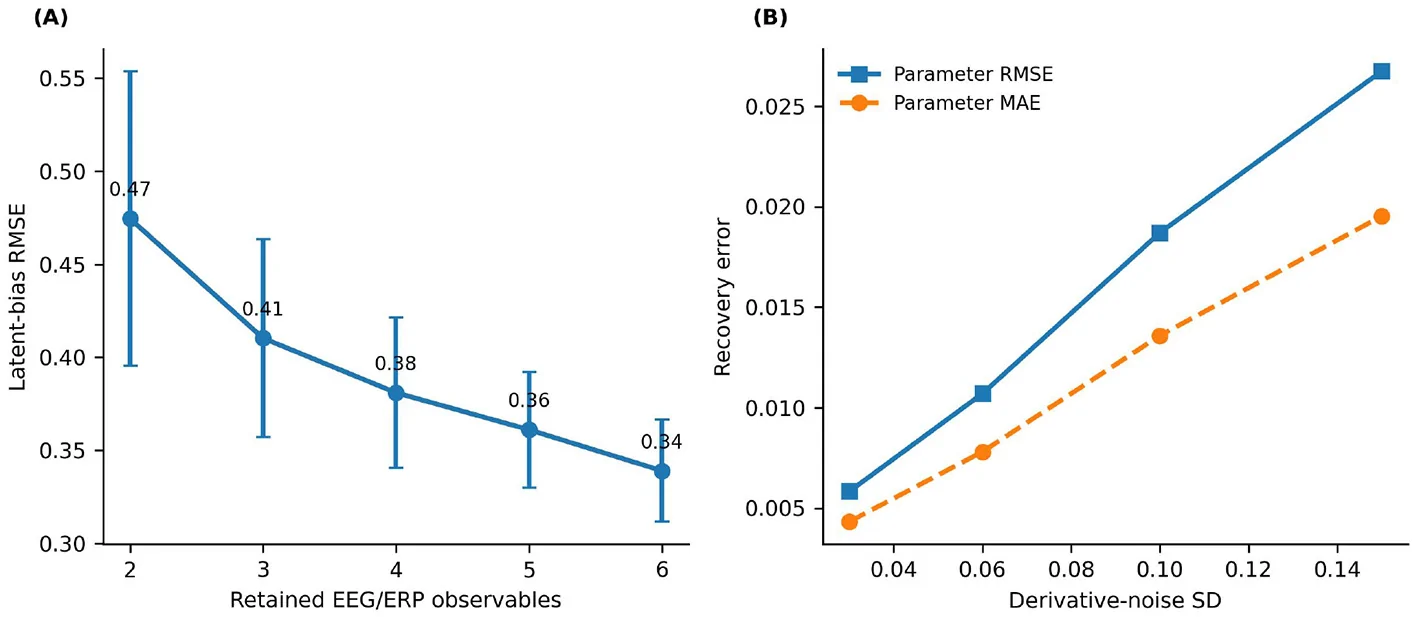
Synthetic **(A)** missing-observable state-reconstruction robustness and **(B)** oracle-state coefficient recovery under increasing derivative noise.

### 3.7 Competing-generator discrimination and full coupled equilibria

The competing-generator experiment separates implementation consistency from structural discrimination. Table 5 reports held-out RMSE averaged over 100 repetitions. When the quartic equilibrium generated the data, the pre-specified quartic candidate achieved RMSE 0.060, compared with 0.497 for the additive model and 0.436 for the linear interaction model, and it was selected by minimum RMSE in all 100 repetitions. Conversely, when the linear interaction generated the data, the interaction candidate was selected in all repetitions. The additive and interaction candidates were nearly tied under the additive generator because the interaction model nests the additive form; this expected ambiguity argues against treating model selection as a universal classifier. The useful conclusion is narrower: under the pre-specified benchmark, the non-linear structure leaves a held-out pattern that simpler candidates do not reproduce.

**Table 5:** Competing-generator benchmark: mean held-out RMSE (100 repetitions).

Generating model	Additive candidate	Interaction candidate	Quartic candidate
Additive linear	0.060	0.060	0.168
Linear interaction	0.133	0.060	0.351
Quartic equilibrium	0.497	0.436	0.060

Full-system equilibrium analysis also narrows the scope of the multistability claim. Table 6 evaluates every equilibrium with the complete Jacobian. At *d* = 0.15, the coupled system changes from one stable equilibrium at *s* = 0 to three equilibria (two stable) by *s* = 1, with the first multistability boundary at *s* = 0.137. At *d* = 0.50 and 0.85, the coupled benchmark already has three equilibria with two locally stable branches at *s* = 0. Thus, restoration of control-arousal feedback shifts the boundary relative to the fixed-*c* scalar slice but preserves the qualitative possibility of stress- and symptom-dependent multistability for the present parameterization. This does not imply that a real participant crosses between branches; switching remains dependent on initial conditions, noise, and the stress trajectory.

**Table 6:** Full coupled (*b, c, r*) equilibrium check.

*d*	*s*	Real equilibria	Locally stable equilibria	First multistability stress
0.15	0	1	1	0.137
0.15	1	3	2	0.137
0.50	0	3	2	0.000
0.50	1	3	2	0.000
0.85	0	3	2	0.000
0.85	1	3	2	0.000

### 3.8 Observation-model conditioning and sensitivity

The nominal standardized loading matrix had condition number 3.85, indicating that the three latent directions were not nearly collinear in this deliberately specified benchmark. Table 7 shows that state-reconstruction RMSE was 0.152 under the nominal map, 0.153 after FAA deletion, 0.232 under 15% loading perturbations, 0.186 with added cross-loadings, and 0.187 under correlated observation noise. Bias-only RMSE remained below 0.11 in these targeted scenarios. The benchmark is therefore not destroyed by modest perturbations, but the loading perturbation causes a clear performance loss and demonstrates that nominal reconstruction accuracy partly depends on correct observation specification. This sensitivity result complements, rather than replaces, the missing-observable analysis in Figure 8.

**Table 7:** Observation-model sensitivity over 100 Monte Carlo repetitions.

Scenario	State RMSE	Bias RMSE
Nominal	0.152	0.074
Drop FAA	0.153	0.079
15% loading perturbation	0.232	0.107
Cross-loading perturbation	0.186	0.104
Correlated noise (ρ = 0.30)	0.187	0.064

*Remark*. The synthetic benchmark is separated from empirical inference. Its strongest conclusion is that equations, code, tables, and figures are internally consistent; the non-linear benchmark can be separated from two simple alternatives under pre-specified simulation conditions; and state reconstruction degrades predictably under observation misspecification. The direct coefficient-recovery experiment is explicitly oracle-state and should not be interpreted as empirical parameter recovery. It cannot establish that depressive symptoms cause the simulated ERP pattern, that a real stressor moves the coupled system across the fixed-control multistability boundary or between attractors, or that the amplitudes are clinically realistic. Those claims require preregistered human data, manipulation checks, and independent replication.

## 4 Discussion

### 4.1 A dynamical interpretation of negative cognitive bias

The computational contribution is the integration of a symptom- and stress-dependent quartic normal form, control-arousal feedback, and a multivariate EEG/ERP observation layer within one falsifiable state-space benchmark, rather than a claim that attractor or state-space methods are themselves new. The main conceptual contribution is to treat stress-related negative cognitive bias as a regime of neural dynamics rather than as a list of component abnormalities. In this view, N2, P3, and LPP changes are temporally distinct projections of a common evolving state. N2 captures early conflict and preferential processing of negative input; P3 reflects context updating and available control; LPP reflects sustained motivated attention. Frontal theta and connectivity provide complementary information about control demand and network coordination. The latent attractor is therefore not identified with any single measure. It is a hypothesis about the joint trajectory needed to explain several measures simultaneously.

The quartic potential gives the model an interpretable non-linear structure. A linear stress model assumes that each unit of stress adds a fixed amount of bias and that recovery is symmetric. The attractor model allows qualitatively different behavior. On the one-equilibrium side of the saddle-node boundary, the fixed-control bias subsystem has a single stationary state. Near the boundary, branch curvature weakens and small perturbations can produce large state changes or slow recovery. Under bistability, identical current stress values can correspond to different bias states depending on prior history. This formulation permits hysteresis as a model prediction rather than establishing it empirically. A future repeated-stressor experiment could therefore distinguish the model from ordinary moderation by testing whether recovery trajectories depend on the direction from which the stress level is approached.

The framework also clarifies why individual EEG markers often have modest reliability or inconsistent diagnostic value. If an ERP is a noisy projection of a multivariate latent state, then its association with symptoms depends on task timing, stress context, observation loading, and the current position in the non-linear landscape. The same person may show a large LPP effect during a stress block but little resting alpha asymmetry. That discrepancy need not be contradictory if the markers load on different state coordinates. Precision-EEG research on individual reliability (Korisky et al., 2026) and time-varying network findings in depression (Yang et al., 2024) support the need to model this context dependence explicitly.

### 4.2 Relation to existing depression EEG research

The model is consistent with several established findings while adding testable cross-measure constraints. Frontal-parietal ERP biases in subthreshold depressive states (Li et al., 2023) motivate a continuous symptom parameter rather than a strict patient-control switch. The P300 meta-analytic literature (Santopetro et al., 2025) motivates a control-sensitive P3 observation but also warns against treating amplitude reduction as specific to depression. LPP evidence in adolescent depression and risk (Dickey et al., 2024) supports sustained emotional-processing markers. Resting theta and PLV findings (Li et al., 2024) motivate oscillatory observables, while emotional-face network studies (Teng et al., 2024) and time-varying connectivity work (Yang et al., 2024) motivate a network state that changes across processing stages.

Stress is modeled as both a direct tilt and a curvature change. ERP evidence indicates that stress can alter reward responsiveness (Pegg and Kujawa, 2024), while recent resting-state synthesis shows that depression is accompanied by heterogeneous oscillatory deviations that should not be reduced to a single spectral marker (Heitmann et al., 2026). The distinction matters. A pure directional field *h* can bias processing without creating a new stable regime, whereas a curvature effect through *a* can change the number and depth of equilibria. Accordingly, future human data should compare nested models: additive stress, directional-field only, curvature only, and full interaction. Model selection should be based on out-of-sample predictive likelihood and recovery of held-out ERP/network features, not solely on in-sample fit.

The model also speaks to self-referential and social appraisal effects. Neurophysiological self-referential processes can predict treatment-related change in adolescent depression (Kim et al., 2025), while socio-emotional appraisal is altered in major depressive disorder (Li et al., 2026). In the proposed framework, self-relevance can enter the directional field as an additional input that selectively deepens the negative basin for personally meaningful stimuli. This extension predicts that stress should have a larger effect on negative self-referential items than equally negative but impersonal stimuli. It also predicts that successful cognitive therapy could act by increasing control coupling γ, decreasing interaction terms *a*_*ds*_ or *h*_*ds*_, or increasing the barrier for transitions into the negative basin.

### 4.3 Empirical tests, model comparison, and falsification

A useful mechanistic theory must be easy to falsify. First, if stress effects on LPP, theta, and behavior are unrelated after accounting for measurement noise, the low-dimensional latent-state hypothesis weakens. Second, if repeated stress produces strictly linear, history-independent recovery at all symptom levels, the attractor interpretation loses support. Third, if the observability Gramian is ill conditioned despite dense EEG, reliable recovery of *b, c, r* is not justified. Fourth, if estimated stress–symptom interactions have opposite directions across replication samples, the current parameterization should be revised rather than protected by flexible *post-hoc* loadings.

The present competing-generator simulation is an internal discrimination check, not empirical validation. A preregistered study should therefore compare the non-linear model against simpler alternatives. Let $M_{0}$ be an additive linear state model, $M_{1}$ be the quartic model without *ds* interaction, and $M_{2}$ be the full model. The expected log predictive density for predictive comparison is defined in Equation 62:

$$\begin{array}{l} ELPD\left(M\right)=\sum_{k=1}^{K}\log p\left(y_{k}^{test}|y^{train},M\right), \end{array}$$ (62)

with the contrast in Equation 63:

$$\begin{array}{l} \Delta ELPD_{21}=ELPD\left(M_{2}\right)-ELPD\left(M_{1}\right). \end{array}$$ (63)

A practically useful full model should improve held-out prediction and reduce structured residuals. It should also retain interpretable parameter estimates across sessions.

### 4.4 Preregistered empirical estimation and replication strategy

A decisive empirical test should estimate subject-specific dynamics without allowing the observation model to absorb every between-person difference. Let **θ**_*i*_ collect the dynamic parameters for participant *i*, including the symptom- and stress-sensitive landscape terms and selected observation loadings. The hierarchical specification in Equation 64 separates population structure from individual variation:

$$\begin{array}{l} \theta_{i}=\mu_{\theta }+L_{\theta }\xi_{i},\;\xi_{i}\sim N\left(0,I\right), \end{array}$$ (64)

where $L_{\theta }L_{\theta }^{⊤}$ is the between-participant covariance. This construction partially pools weakly identified individual parameters while preserving continuous symptom variation. It also avoids the artificial precision that would result from fitting every participant independently when the number of stress blocks or artifact-free trials is limited.

For observed EEG/ERP sequences, estimation should be based on the joint state-space likelihood rather than on component-wise regressions alone. If ***y***_*ik*_ denotes the observation vector for participant *i* and trial or time index *k*, the marginal likelihood is given by Equation 65:

$$\begin{array}{l} p\left(y_{i}|\theta_{i}\right)=\int p\left(z_{i0}|\theta_{i}\right)\prod_{k=1}^{K_{i}}p\left(z_{ik}|z_{i,k-1},\theta_{i}\right)\\ p\left(y_{ik}|z_{ik},\theta_{i}\right)dz_{i,0:K_{i}}. \end{array}$$ (65)

For the non-linear model, this integral may be approximated with an extended or unscented Kalman filter when posterior mass is concentrated near one branch, and with sequential Monte Carlo near strongly non-Gaussian transitions. The primary diagnostic should not be in-sample fit. Posterior predictive checks should test whether the fitted model jointly reproduces ERP amplitude distributions, cross-feature covariance, stress recovery trajectories, and the frequency of transitions between negative and flexible states.

Measurement invariance across sessions and experimental conditions is especially important. Apparent changes in *b* can be confounded with changes in the loading matrix if electrode impedance, preprocessing, vigilance, or reference configuration systematically alter the mapping from latent state to observables. A parsimonious session-specific model is specified in Equation 66:

$$\begin{array}{l} H_{j}=H_{0}+\Delta H_{j},\;{\left\|\Delta H_{j}\right\|}_{F}\leq \varepsilon_{H}, \end{array}$$ (66)

where *j* indexes session, and ε_*H*_ is a preregistered tolerance or regularization scale. The stress-bias interpretation would be strengthened if the principal dynamic parameters remain stable when modest session-specific loading deviations are permitted. Conversely, a nominal stress effect that disappears after allowing plausible Δ***H***_*j*_ should be interpreted as measurement-sensitive rather than as evidence of altered attractor geometry.

The confirmatory analysis should distinguish parameter recovery, predictive adequacy, and clinical association. Parameter recovery asks whether known simulated parameters can be reconstructed under the exact planned design. Predictive adequacy asks whether the non-linear model improves held-out prediction relative to additive and interaction-only alternatives. Clinical association asks whether estimated dynamic quantities vary with symptom severity or prospectively predict change. A preregistered participant-level stress-sensitivity estimand is defined by Equation 67:

$$\begin{array}{l} \psi_{i}={\frac{\partial b_{i}^{*}\left(s\right)}{\partial s}|}_{s=s_{0}}, \end{array}$$ (67)

and Equation 68 specifies its symptom association without dichotomizing severity:

$$\begin{array}{l} \psi_{i}=\alpha_{\psi }+\beta_{\psi }d_{i}+q_{i}^{⊤}\gamma_{\psi }+\epsilon_{i}, \end{array}$$ (68)

where ***q***_*i*_ contains preregistered nuisance variables such as age, medication status, and baseline arousal when clinically appropriate. The key test is the uncertainty interval for β_ψ_ together with out-of-sample calibration, not merely whether a conventional significance threshold is crossed.

Replication should preserve the model's falsifiability. An initial study can estimate plausible ranges of dynamic parameters and measurement loadings, whereas a second independent cohort should hold the primary model structure fixed and evaluate pre-specified predictive contrasts. Cross-site validation should additionally test whether the same latent coordinates remain observable under different EEG systems and preprocessing pipelines. Empirical support for the framework would therefore require convergent support at three levels: stable measurement of the proposed observables, recoverable and reproducible latent dynamics, and improved prediction of stress-related cognitive bias beyond simpler linear models. This staged strategy limits researcher degrees of freedom and makes a failed replication diagnostically useful rather than an invitation to redefine the latent construct after seeing the data.

### 4.5 Limitations

The most important limitation is deliberate: there are no human participants in the present study. Consequently, the simulated ERP amplitudes, symptom interactions, repeated-sampling intervals, model-selection frequencies, reconstruction errors, and coefficient-recovery errors cannot be interpreted as clinical estimates or evidence that the dynamic parameters are practically identifiable from real EEG. In particular, the EEG/ERP observation loading structure remains theoretically specified and uncalibrated against human EEG datasets; its empirical adequacy must be assessed in future studies using human recordings. The study provides an original computational model and reproducible benchmark that are ready for empirical testing. A future empirical study must separately report recruitment, diagnostic assessment, stress manipulation, ethics approval, electrode montage, preprocessing quality, exclusions, trial counts, and adverse-event monitoring.

Second, a three-dimensional latent state is a simplification. Negative cognitive bias involves multiple subprocesses, including attentional capture, disengagement, interpretation, autobiographical memory, reward learning, and rumination. The current coordinates are chosen for low-dimensional theoretical clarity and local state observability, not as an exhaustive neural ontology or a guarantee of parameter identifiability. Third, scalp EEG has limited spatial specificity and connectivity measures can be inflated by common sources. Source-space or leakage-corrected connectivity should therefore be evaluated. Fourth, depression is heterogeneous. Medication, anxiety, sleep, trauma, age, and recurrent episode history may change either the dynamic parameters or observation loadings.

Finally, the quartic potential should be viewed as a minimal normal form around a possible tipping region. It is attractive because it yields explicit equilibria, barriers, and a bifurcation criterion. It is not asserted that cortical activity literally minimizes a scalar psychological energy. Competing non-linear forms, switching state-space models, and neural-mass models may fit future data better. The value of the current formulation lies in producing precise, testable predictions with a small number of parameters rather than in claiming a unique biophysical mechanism.

*Remark*. The framework is intended to change the unit of explanation from “a significant ERP component” to “a constrained dynamical state supported by several measurements.” That shift raises the evidential bar. A future dataset should show not only a stress-by-symptom effect but also coherent temporal, oscillatory, network, and behavioral consequences, recoverable parameters, and better out-of-sample performance than linear alternatives. Failure on any of these dimensions would be informative and should guide model revision.

### 4.6 Conclusion and future work

This simulation-based study proposed a non-linear EEG/ERP framework for stress-related negative cognitive bias. Theoretical analysis establishes bounded stochastic dynamics, a conditional scalar multistability criterion, stable-branch sensitivity, and a sufficient local state-observability condition. Reviewer-driven extensions show that (i) the full coupled equilibrium system preserves multistability over the benchmark parameter range, although its boundary shifts relative to the fixed-control slice; (ii) the pre-specified non-linear generator is distinguishable from additive and interaction-only alternatives in held-out synthetic data; (iii) FAA follows the same model-implied moderation logic as the other observation channels; and (iv) state reconstruction degrades under plausible loading perturbations. The direct drift-coefficient experiment remains an oracle-state implementation check. Because no human EEG was used, all quantitative outputs are methodological demonstrations and falsifiable hypotheses rather than clinical findings or evidence of empirical parameter identifiability.

Future work will proceed in three directions. First, a preregistered longitudinal EEG study should collect repeated baseline, acute-stress, and recovery blocks across a continuous range of depressive symptoms, enabling direct tests of tipping, hysteresis, and recovery time. Second, source-resolved EEG/MEG and autonomic measures should be integrated to determine whether the latent coordinates have reproducible network and physiological correlates beyond scalp amplitude features. Third, hierarchical Bayesian estimation should be developed for person-specific parameters and prospective prediction, with external validation across sites, EEG systems, age groups, and treatment contexts.

## Data Availability

The original contributions presented in the study are included in the article/supplementary material, further inquiries can be directed to the corresponding author.
